# Placenta percreta causing spontaneous uterine rupture and intrauterine fetal death in an unscared uterus: A case report

**DOI:** 10.1016/j.ijscr.2019.10.039

**Published:** 2019-11-01

**Authors:** J.T. Enebe, I.J. Ofor, I.I. Okafor

**Affiliations:** a Department of Obstetrics & Gynaecology, Enugu State University of Science and Technology College of Medicine/Teaching Hospital, Parklane, Enugu, Nigeria; b Department of Obstetrics & Gynaecology, Enugu State University of Science and Technology Teaching Hospital, Parklane, Enugu, Nigeria

**Keywords:** Uterine rupture, Plcacenta percreta, Maternal morbidity, Intrauterine fetal death, Case report

## Abstract

**Introduction:**

Placenta percreta is a rare; a life-threatening disorder of placentation and one of the components of the placenta accreta spectrum. It can lead to uterine rupture, an obstetric catastrophe that can be associated with increased maternal and fetal morbidity and mortality.

**Presentation of case:**

We present an unusual case of spontaneous uterine rupture due to placenta percreta in an unscarred uterus of a multiparous woman leading to spontaneous intrauterine fetal death. She presented with hypovolaemic shock following spontaneous rupture of the uterus and subsequent intra-peritoneal bleeding.

**Discussion:**

Uterine rupture occurs commonly in a scarred uterus from some form of trauma or injudicious use of oxytocics. However, uterine rupture occurring in the absence of prior scar or use of oxytocics is a rarity. Placenta percreta is an unusual cause of uterine rupture and subsequent intra-uterine fetal death. Placenta percreta occurs when the uterine wall is invaded by the placenta up to the level of the serosa. A high index of suspicion and thorough review of the patient is required for making this diagnosis. Misdiagnosis is associated with dare consequences of increased maternal morbidity and mortality.

**Conclusion:**

Placenta percreta is a rare disorder of placentation that can cause uterine rupture which can easily be misdiagnosed. Prompt diagnosis and institution of the appropriate care can help prevent catastrophic outcomes as demonstrated in the case reported.

## Highlights

• Placenta percreta causing hemoperitoneum and intrauterine death can easily be misdiagnosed.
• Uterine rupture is rare in unscarred uterus and in absence of trauma.
• Placenta percreta is an unusual cause of uterine rupture.
• Placental percreta is an unusual cause of heamoperitoneum and intra-uterine death.

## 1 Introduction

Placenta percreta is a rare disorder of placentation and one of the components of the placenta accreta spectrum (morbidly adherent placenta spectrum). It is a life-threatening condition for both the mother and the fetus. Placenta percreta has the potential to cause adverse events that could occur in the antepartum, intrapartum and postpartum periods [1,2]. One of these rare adverse events is uterine rupture which is an obstetric catastrophe with high maternal and perinatal morbidity/mortality [[1], [2], [3]].

We present an unusual case of spontaneous uterine rupture in an unscarred uterus due to placenta percreta in a multiparous woman. The SCARE criteria were utilized in the report of this case [4].

## 2 Presentation of case

**Fig. 1:**
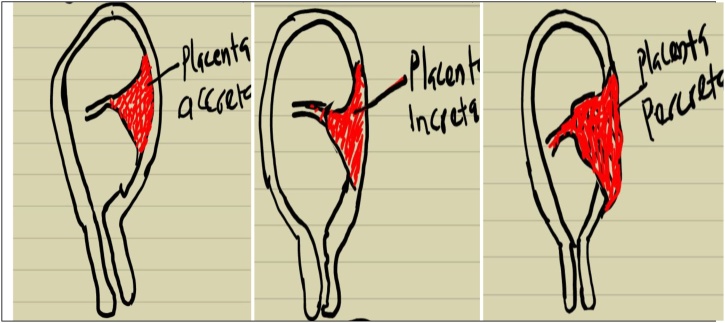
Morbidly adherent placenta spectrum.

**Fig. 2:**
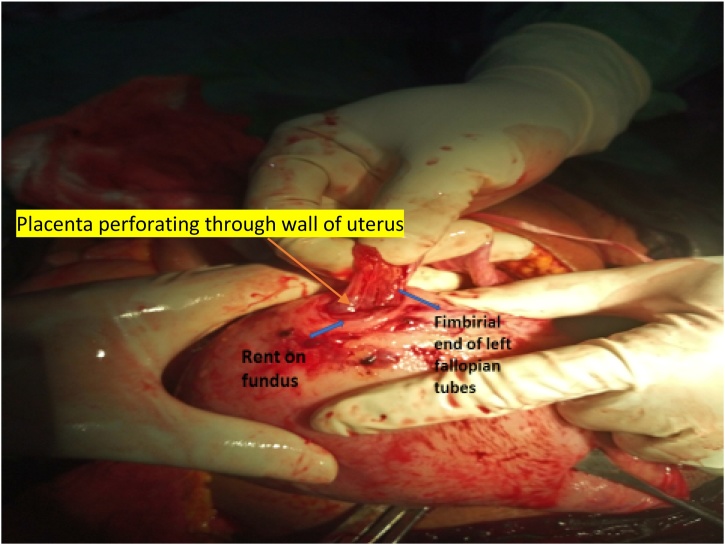
Showing uterus with rupture and placenta jutting out of the fundus attaching firmly to the fimbyial end of the right fallopian tube causing haemoperitoneum.

**Fig. 3:**
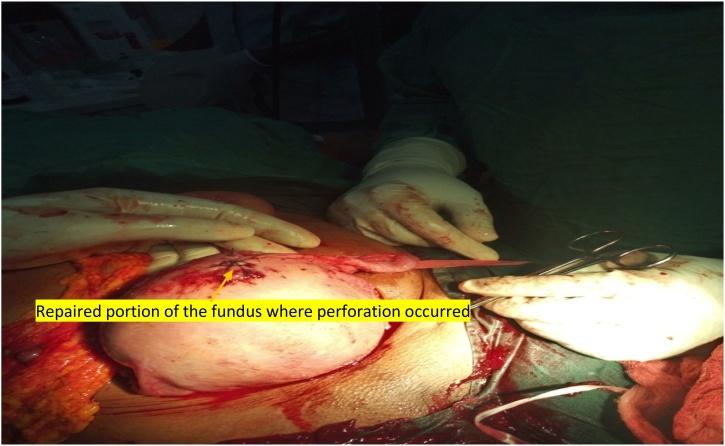
Showing fundus of the uterus after repair of site of placental perforation and uterine rupture.

An unbooked 34-year-old woman, who was G_5_P_3_^+1^ with three living children was rushed into the labour ward due to fainting spells of 4 h duration. There was an associated abdominal pain. She was not a known hypertensive. There was no history of bleeding per vaginam, sentinel bleeds, or abdominal trauma. However, she had prior history of termination of pregnancy via dilatation and curettage 10 years prior to her presentation with no significant post abortion complications (Fig. 1, Fig. 2, Fig. 3).

On presentation, she was markedly pale, the pulse was feeble and barely palpable, and the blood pressure was not recordable. The abdomen was markedly tender and about 36 weeks size. The fetal heart tones were not recordable with a hand held doppler. Her vaginal examination was unremarkable. An initial diagnosis of concealed abruptio with intrauterine fetal death was made. Intravenous access was secured with two size 16 canula and samples were collected for urgent packed cell volume, grouping and cross matching of blood. Normal saline was rushed through one of the vascular accesses while compatible blood was cross-matched. Antishock garment was immediately applied as appropriate. An urgent bedside ultrasound scan confirmed intrauterine fetal death (IUFD) of a 33-week fetus in breech presentation. A fundal placenta with evidence of echorich particulate fluid in the peritoneum (haemoperitoneum) was also noted.

The patient’s urgent packed cell volume (PCV) was 13% and compatible blood transfusion was commenced immediately while the patient was prepared for emergency laparotomy. Intra-operative findings revealed massive haemoperitoneum of about three litres. A rent was noted in the fundus of the uterus with placental tissue jutting out of the fundal aspect of the uterus and mildly oozing blood. This rent communicated with the endometrial cavity. The fimbrial end of the right fallopian tube also attached to both the rent site and the placenta necessitating a right partial salpingectomy. A female fresh stillborn neonate that weighed 2.1 kg in breech presentation was delivered.

The rent in the uterus was repaired with appropriate sutures. She received a total of 3 units of whole blood at the end of the surgery. Her immediate post operation condition was stable. She received further two units of blood in the ward. Her post transfusion PCV was 29%. She was adequately counselled on the surgery that was done and her future fertility potentials. She was also counseled on the need for spacer contraception and importance of booking early for antenatal care in her subsequent pregnancies. She was discharged home on the 6^th^ day after surgery in a stable condition.

## 3 Discussion

Placenta percreta occurs when the placenta abnormally attaches and invades the myometrium and serosa [5]. It occurs due to abnormal development of the decidua basalis and previous injury to the uterine wall layers [5]. The incidence of placenta percreta is about 1 in 533 [6]. Placenta percreta is the rarest form of the placenta accreta spectrum and believed to represent 5–7% of all abnormal placentation [6].

Uterine rupture usually occurs in a scarred uterus following some trauma. However, the uterus may also get ruptured without a prior scar. Placenta percreta is an unusual cause of uterine rupture and subsequent antepartum haemorrhage. It has been documented to cause uterine rupture in any trimester [[1], [2], [3]]. As previously reported by Khandaker [1], the index patient had uterine rupture in the third trimester.

Risk factors for the occurrence of placenta percreta include previous uterine surgeries (caesarean delivery, myomectomy and metroplasty) [1,2], dilatation and curettage for abortion and previous history of manual removal of placenta [7]. Also, whole body radiation therapy for medical conditions like childhood leukemias has been reported as an uncommon cause of placenta percreta [3]. The index patient had a prior history of dilatation and curettage ten years prior to presentation. She has had two previous uncomplicated pregnancies prior to the index pregnancy. Placenta percreta has also been reported to occur without any known predisposing factors [8,9]. The diagnosis can often be missed due to preference for diagnosing more common causes of abdominal pain and anaemia in pregnancy such as concealed abruptio placentae as reported in the index case and case reported by Vyjayanthi and colleagues [7]. Delay in diagnosing the cause may lead to increased maternal and fetal morbidity and even mortality. We had a neonatal mortality in our case and a near miss for the mother. Most cases of placenta percreta causing uterine rupture in the third trimester usually result in perinatal mortality. However, in cases where early diagnosis is made and there is mild haemorrhage, fetal salvage may be possible [1].

Management of placenta percreta causing uterine rupture depends on the clinical scenario. If the entire placenta is morbidly adherent, hysterectomy should be performed [2,3,7,9]. However, if the placenta can be delivered easily, the rent on the uterus can be repaired as was done in the index patient and in the case reported by Khandaker [1]. Laparoscopic repair of the rent in stable cases has been attempted in the second trimester, however in that case report, eventual hysterectomy was performed when evacuation of the products of conception failed [10].

## 4 Conclusion

Placenta percreta is a rare disorder of placentation that can cause uterine rupture and this diagnosis can be easily missed. Prompt diagnosis and management will avert catastrophic maternal and perinatal outcomes as demonstrated in the case reported.

## Sources of funding

This study was fully funded by the authors. There was no external source of funding in this research.

## Ethical approval

Ethical approval was obtained from Ethic and Research Committee of the Enugu State University of Science and Technology Teaching Hopsital, Parklane Enugu with reference number: ESUTHP/C-MAC/RA/034/Vol.11/51 and dates 22nd August 2018.

## Consent

Written informed consent was obtained from the patient for publication of this case report and accompanying images. A copy of the written consent is available for review by the Editor-in-Chief of this journal on request.

## Author’s contribution

Enebe Joseph Tochukwu - Concept and design of study, drafting the article and final approval of the submission.

Ofor Ifeanyichukwu J - Concept and design of study, drafting the article and final approval of the submission.

Okafor Innocent I - Concept and design of study, drafting the article and final approval of the submission.

## Registration of research studies

N/A.

## Guarantor

Enebe Joseph Tochukwu.

## Provenance and peer review

Not commissioned, externally peer-reviewed.

## Declaration of Competing Interest

There is no conflict of interest to declare.
